# Long-Term Renal Outcomes Among COVID-19 ICU Survivors Admitted With Acute Kidney Injury

**DOI:** 10.7759/cureus.99757

**Published:** 2025-12-21

**Authors:** Rocio Rimachi, Hassane Ndjimi, Keitiane Kaefer, Antonella Cudia, Pierre Foulon, Daniel De Backer

**Affiliations:** 1 Intensive Care Unit, CHIREC Hospitals, Brussels, BEL; 2 Department of Intensive Care, Université Libre de Bruxelles, Brussels, BEL; 3 Department of Intensive Care, Erasme University Hospital, Brussels, BEL; 4 Intensive Care Unit, Brugmann University Hospital, Brussels, BEL

**Keywords:** acute kidney injury, covid-19, long-term outcomes, renal replacement therapy, severe respiratory failure

## Abstract

Introduction

Acute kidney injury (AKI) is associated with poor outcomes in intensive care unit (ICU) patients with COVID-19. The need for renal replacement therapy (RRT) was frequently reported among these patients. However, the long-term prognosis of new-onset AKI detected on ICU admission is not well defined, in opposition to AKI occurring at any time during ICU stay. We evaluated the long-term outcomes of early-AKI (present on admission) in COVID patients requiring ICU during the pandemic; identified AKI risk factors and the impact of RRT on survival and renal outcomes (short and long term).

Methods

We conducted an observational study with retrospective analysis charts of COVID-19 adult patients consecutively admitted to the ICU at CHIREC Hospital, Brussels, Belgium, between March 2020 and April 2021. AKI criteria were assessed using KDIGO (Kidney Disease Improving Global Outcomes) criteria at baseline and daily during ICU stay. The data are reported at admission, at the time of the worst AKI stage, at ICU discharge, and at long-term follow-up 3.5 years after ICU discharge.

Results

Among the 247 COVID-19 patients admitted to the ICU, 236 were included in the analysis. Early AKI was detected in 87 (37%) patients. Age, acute respiratory distress syndrome, hypertension, diabetes, and obesity were significantly associated with the development of AKI. Sixty (69%) patients were in stage 1, nine (10%) in stage 2, and 18 (21%) in stage 3. Among patients with early AKI, 52 survived to the ICU, and 47 were available for long-term follow-up. ICU mortality was higher in patients with AKI stage 3 (66%) than those with AKI stage 1 (32%), and this mortality increased with AKI progression and the need for renal replacement therapy (RRT). In the early-AKI group, 22 patients (25%) required RRT during ICU stay, two (9%) received RRT at admission, while 20 (90%) received RRT during ICU stay. Among these 22 patients, 19 (86%) died in the ICU, while three (13%) survived to follow-up and were able to discontinue RRT.

Conclusions

Early AKI is common in COVID-19 ICU patients with acute respiratory failure and is associated with increased ICU length of stay compared with that of no-AKI patients. Patients with poor progression of AKI during their ICU stay and those with RRT requirements have a high mortality rate; however, survivors generally experience sufficient renal recovery to discontinue RRT. Early-AKI survivors without RRT requirements show excellent recovery of renal function.

## Introduction

According to the latest World Health Organization (WHO) report, severe acute respiratory syndrome coronavirus 2 (SARS-CoV-2) has killed 7,103,185 people [1]. During the initial waves of SARS-CoV-2 infection, most attention was focused on respiratory issues, as many severe cases progressed to acute respiratory distress syndrome (ARDS), and patients were hospitalized in intensive care units (ICUs) mostly for respiratory support. However, acute kidney injury (AKI) may also occur in coronavirus disease 2019 (COVID-19) patients admitted to the ICU. The pathophysiology of AKI has not been entirely elucidated and may involve both direct and indirect mechanisms. SARS-CoV-2 has a remarkable kidney tropism that directly infects the kidneys by binding to the kidney angiotensin-converting enzyme 2, leading to AKI [2]. Notably, acute tubular injury has been reported during autopsies [3]. In addition, viral infections and the associated immune response may contribute to endothelial injury, microvascular thrombi, local inflammation, and immune cell infiltration [4]. Organ dysfunction during the acute phase of severe COVID-19 can persist or may lead to an inability to recover over a prolonged and arduous ICU stay. Numerous studies [5-7] assessing the impact of AKI have included patients who developed AKI at any point during their ICU stay. There is limited information on long-term patients and renal outcomes of patients admitted to the ICU with COVID presenting AKI already on ICU admission (early AKI).

In this observational study, we evaluate long-term renal outcomes, specifically examining the effect of renal replacement therapy (RRT) in COVID-19 patients admitted during the first and second waves in Belgium with moderate to severe respiratory failure and early AKI. The secondary objective was to identify patients' characteristics and risk factors associated with adverse renal outcomes.

## Materials and methods

This study was conducted in the Department of Intensive Care at CHIREC Hospital, Brussels, Belgium, which serves critically ill adult patients. The ICU, initially with 30 beds, expanded to 54 beds during the peak of the COVID wave. The hospital's ethical review board approved study 2022-39 and waived the need for informed consent, as this was an epidemiological study without intervention. The study aligns with the Declaration of Helsinki principles.

Participants and procedure

We retrospectively analyzed the charts of all adult COVID-19 patients consecutively admitted to the ICU from March 2020 to April 2021 and followed up for approximately 3.5 years post-ICU discharge, with a follow-up duration ranging from 40 to 49 months. We included patients with confirmed COVID-19, defined by positive SARS-CoV-2 reverse transcription polymerase chain reaction results from nasopharyngeal swabs or hypoxemia associated with a CT scan consistent with the COVID-ARDS pattern [8].

Exclusion criteria were an age of less than 16 years, non-confirmed COVID-19 respiratory failure, chronic kidney disease (CKD) prior to hospitalization, and inability to follow up. 

Data collection

Data were collected retrospectively from medical records. All patient information was anonymised, and each individual was assigned a unique study number to ensure confidentiality. Demographics and medical data included age, gender, diagnosis, comorbidities, total ICU, and hospital length of stay (LOS), the Sequential Organ Failure Assessment (SOFA) score [9], and major therapeutic interventions (mechanical ventilation, continuous renal replacement therapy (RRT), extracorporeal membrane oxygenation (ECMO), and tracheotomy).

AKI definition and staging

AKI was defined according to the Kidney Disease Improving Global Outcomes (KDIGO) criteria [10]. The AKI 2012 helps identify patients with varying severity of AKI: stage 1(AKI-1), stage 2 (AKI-2), and stage 3 (AKI-3). Baseline creatinine was defined as the lowest creatinine measurement within the 6six months prior to admission; in the absence of any prior blood test, a baseline GFR of >75 ml/min/m^2^ was assigned to back-calculate serum creatinine levels [11,12]; No-AKI is considered if baseline was <0.7 mg/dl (females) and 0.9 mg/dl (males) [12]. Early AKI was defined as AKI (at any stage) already present at ICU admission. AKI recovery (Ar) is defined according to the KDIGO criteria as return of serum creatinine to <1.5 x baseline and the absence of AKI stage 1, 2, or 3 criteria.

AKI evolution

The AKI criteria were assessed at baseline and daily throughout the ICU stay. Data are reported at four representative times: at admission, at the time of the worst AKI stage during the ICU stay, at ICU discharge, and during the long-term follow-up period (the last creatinine value available was closest to 42 months post-ICU discharge). The initiation of RRT was determined according to established guideline criteria [10,13,14], taking into account individual characteristics and guided by clinical judgement. For long-term data, surviving patients with early AKI were followed and evaluated for renal recovery using medical records of laboratory tests and consultations after hospital discharge, supplemented by interviews with their family doctors when early-AKI patients did not return to the hospital for medical follow-up after discharge. During the COVID-19 pandemic, telephone interviews were frequently employed as an alternative means of data collection. 

Statistical analysis

Continuous variables were reported as means ± standard deviation (SD) for normally distributed data and as medians with interquartile ranges for skewed data. Categorical variables were summarised as counts and percentages (%). Baseline characteristics were compared between early-AKI and no-AKI patients using Fisher’s exact test or the Mann-Whitney U test, as appropriate. AKI progression over time was presented as an alluvial plot. Statistical significance was defined as p< 0.05, and all tests were two-tailed. Statistical analyses were performed using the IBM SPSS Statistics for Windows, Version 27.0.0 (released 2019, IBM Corp., Armonk, NY).

## Results

**Figure 1:**
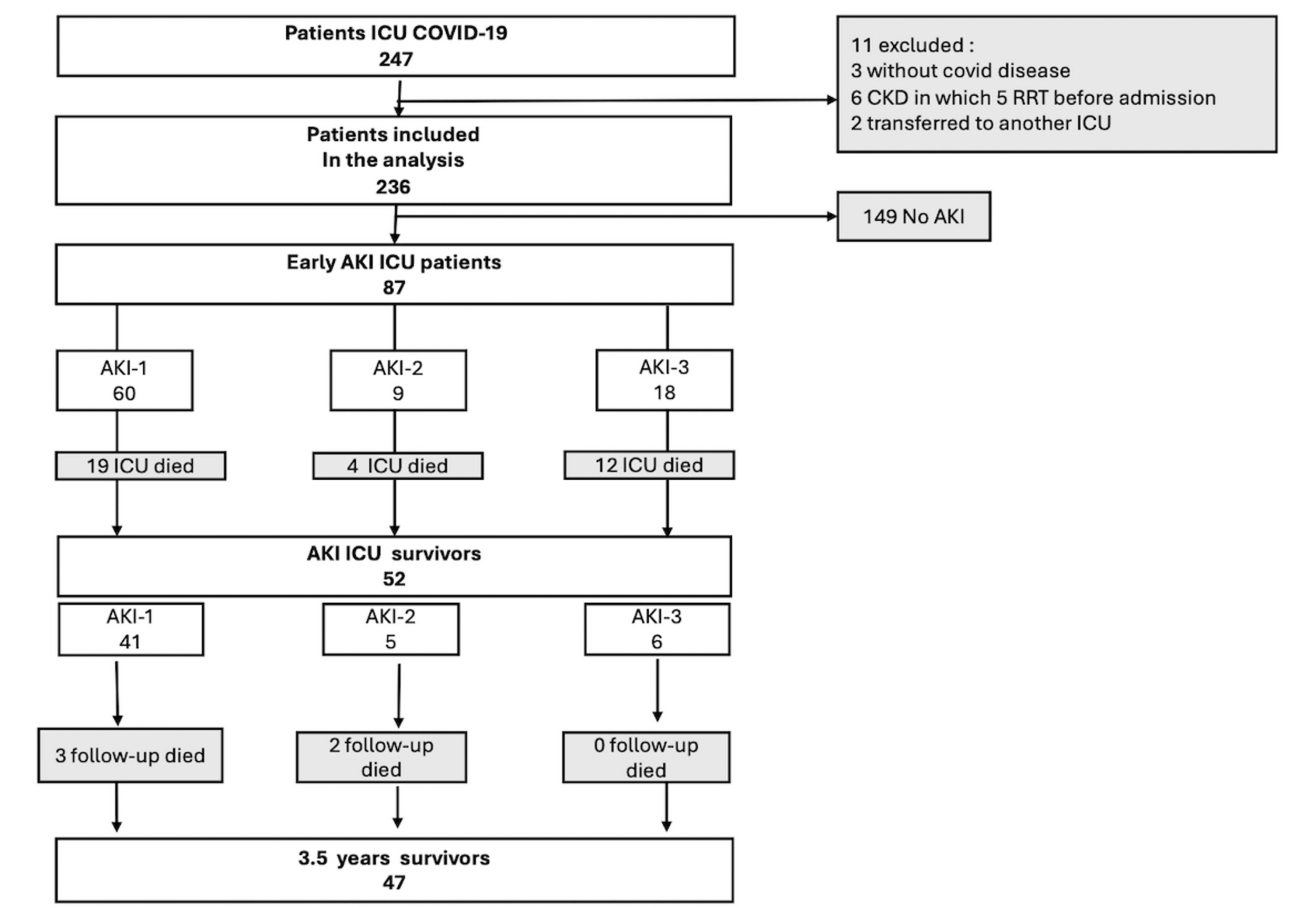
Patients’ selection process and follow-up CKD: chronic kidney disease; RRT: renal replacement therapy Figure created by the authors with MS Word and PowerPoint (Microsoft Corp., USA)

A total of 247 COVID-19 patients were admitted to the ICU between March 2020 and April 2021. Of the 236 patients included in the analysis, 11 were excluded because of the absence of signs of COVID infection in three patients (admitted for traumatic brain injury, gastrointestinal bleeding, and suicide attempt), six with chronic renal failure in which five required chronic hemodialysis, and two patients were transferred to another hospital following the instructions of the Ministry of Health, leading to a loss of follow-up, as shown in Figure 1. 

Patients’ characteristics at baseline

The 236 patients were divided into early-AKI (87 patients) and no-AKI (149 patients) groups. Patient characteristics are detailed in Table 1. Most patients were young, with a median age of 56.3 years, predominantly male, were older, had more comorbidities, had a higher admission SOFA score, and underwent major therapeutic interventions such as mechanical ventilation, ECMO, and RRT for a longer duration during their ICU stay. Apart from patients with CKD, none of the patients included in the study had previously experienced an episode of AKI. The most frequent comorbidities in both groups were hypertension, obesity, and diabetes mellitus. The median ICU LOS was 13 (5-37.2) days, higher in the early-AKI group than in the no-AKI group (20 (7-46) vs. nine (4-26.2) days, respectively; p = 0.002). ICU survival was significantly higher in patients with no-AKI than in those with early-AKI (p < 0.001).

**Table 1:** Patients' characterisitics BMI: body mass index; HTA: hypertension arterial; SOFA: Sepsis-Related Organ Failure Assessment; ECMO: extracorporeal membrane oxygenation; RRT: renal replacement therapy; ICU LOS: intensive care unit length of stay; HOSPITAL LOS: hospital length of stay; IQR: interquartile range; n: sample size; SD: standard deviation; OR: odds ratio; CI: confidence interval Statistically significant data p < 0.05: ° Mann-Whitney U test, * Fisher's exact test

Variable	Overall	Early-AKI	No-AKI	° U value	p-value	
	N = 236	N = 87	n = 149	*OR (CI 95%)		
Age, years (mean ±SD)	56 ± 15.2	59.8 ± 13.5	54.2 ± 15.8	U = 7975.0	p < 0.004°	
BMI, kg/m^2 ^( mean ±SD)	25.4 ± 6.4	28.2 ± 7	27.6 ± 6	U = 7124.0	P = 0.204°	
SOFA score (mean ±SD)	3.1 ± 2.6	5.0 ± 3.1	3.2 ± 2	U = 8813.5	p < 0.001°	
ICU LOS (days), median (IQR)	13 (5.0-37.2)	20 (7.0-46)	9 (4.0-26.2)	U = 8058.5	p = 0.002°	
Hospital LOS (days), median (IQR)	6 (2.0-15)	12(4.3-22.5)	7 (3.0-17.0)	U = 5400.0	p = 0.044°	
Diabetes	54 (23%)	29 (33%)	25 (17%)	2.48 (1.34–4.61)	p = 0.00595*	
HTA	109 (46.1%)	54 (62%)	55 (37%)	2.80 (1.62–4.83)	p = 0.000244*	
Cardiac failure	18 (7.5%)	13 (15%)	5 (3%)	5.06 (1.74–14.73)	p = 0.00188*	
COPD	17 (7%)	11 (13%)	6 (4%)	3.45 (1.23–9.69)	p = 0.01836*	
Asthma	20 (8.3%)	11 (13%)	9 (6%)	2.25 (0.87–5.86)	p = 0.09245*	
Cancer	17 (7%)	7 (8%)	10 (7%)	1.22 (0.44–3.37)	p = 0.79543*	
Neuromuscular disease	8 (3.3%)	5 (6%)	3 (2%)	2.97 (0.69–12.8)	p = 0.14851*	
Autoimmune disease	8 (3.3%)	2 (2%)	6 (4%)	0.56 (0.12–2.65)	p = 0.71365*	
Mechanical ventilation	142 (60%)	65 (75%)	77 (51%)	2.76 (1.55–4.94)	p = 0.000548*	
RRT	25 (11%)	22 (25%)	3 (2%)	12.27 (4.14-37.0)	p = 0.0000002*	
ECMO	20 (8.5%)	14 (16%)	6 (4%)	4.57 (1.69–12.39)	p = 0.00266*	
ICU survival	189 (80%)	52 (60%)	137 (92%)	5.22 (2.75–9.92)	p = 0.00000024*	

Incidence and trajectories of early AKI

Among the 87 patients included in early-AKI, 60 (69%) were in AKI stage 1 (AKI-1), nine (10%) had AKI stage 2 (AKI-2), and 18 (21%) had AKI stage 3 (AKI-3). Table 2 presents the baseline characteristics of early-AKI patients categorized by the AKI severity stage. The median age was comparable across the three groups, which were male predominant and had similar comorbidities. The SOFA score was elevated in the AKI-2 and AKI-3 groups, and these groups also experienced longer ICU stays. The mean creatinine levels on admission were 1.2 mg/dL in AKI-1, 2.24 mg/dL in AKI-2, and 3 mg/dL in AKI-3. Marked oliguria was noted on admission in all stages. The RRT involved continuous venovenous hemodiafiltration (CVVHDF) with citrate anticoagulation. The median duration of RRT was 25 days.

**Table 2:** Baseline characteristics of early-AKI patients BMI: body mass index; HTA: hypertension arterial; SOFA: Sepsis-Related Organ Failure Assessment; ECMO: extracorporeal membrane oxygenation; RRT: renal replacement therapy; ICU LOS: intensive care unit length of stay; HOSP LOS: hospital length of stay

Variables	Early-AKI	AKI-1	AKI-2	AKI-3 (n = 18)
	(n = 87)	(n = 60)	(n = 9)	
Age, years (mean ± SD)	59.8 (13.5)	57.7 (13.2)	62.3 (12)	65 (14)
Male (n, %)	61 (69%)	40 (67%)	6 (67%)	15 (84%)
BMI , kg/m^2^	28.2 ± 7	29.2 ± 7.26	35 ± 9.9	26.3 ± 6.3
(mean±SD)				
Diabetes (n, %)	29 (33%)	18 (30%)	5 (55.5%)	6 (33%)
HTA (n, %)	54 (62%)	37 (61.6%)	5 (55.5%)	12 (67%)
SOFA score (mean ± SD)	5 ± 3.1	4.3 ± 2.7	7.4 ± 3.7	6 ± 3
Mechanical ventilation	65 (75%)	40 (66.6%)	9 (100%)	16 (88%)
(n, %)				
ECMO (n, %)	14 (16%)	7 (12%)	1(11%)	6 (32%)
Creatinine, mg/dL (mean ± SD)	1.6 ± 0.8	1.2 ± 0.2	2.20 ±.3	2.9 ± 0.6
RRT at admission (n, %)	2 (2%)	0	0	2 (11%)
RRT during ICU stay (n, %)	20 (23%)	12 (20%)	1 (11%)	7 (39%)
ICU LOS (days), median (IQR)	36.4 (41.7)	27.3 (30)	62 (60)	49.6 (53)
Hospital LOS (days), median (IQR)	13.2 (19.5)	11.2 (16.2)	30 (37.9)	12.5 (32)
ICU mortality	35 (40%)	19 (32%)	4 (44%)	12 (66%)
(n, %)				
Survival at 3.5 years among ICU survivors (%)	47 (54%)	38 (65%)	3 (33%)	6 (33%)

**Figure 2:**
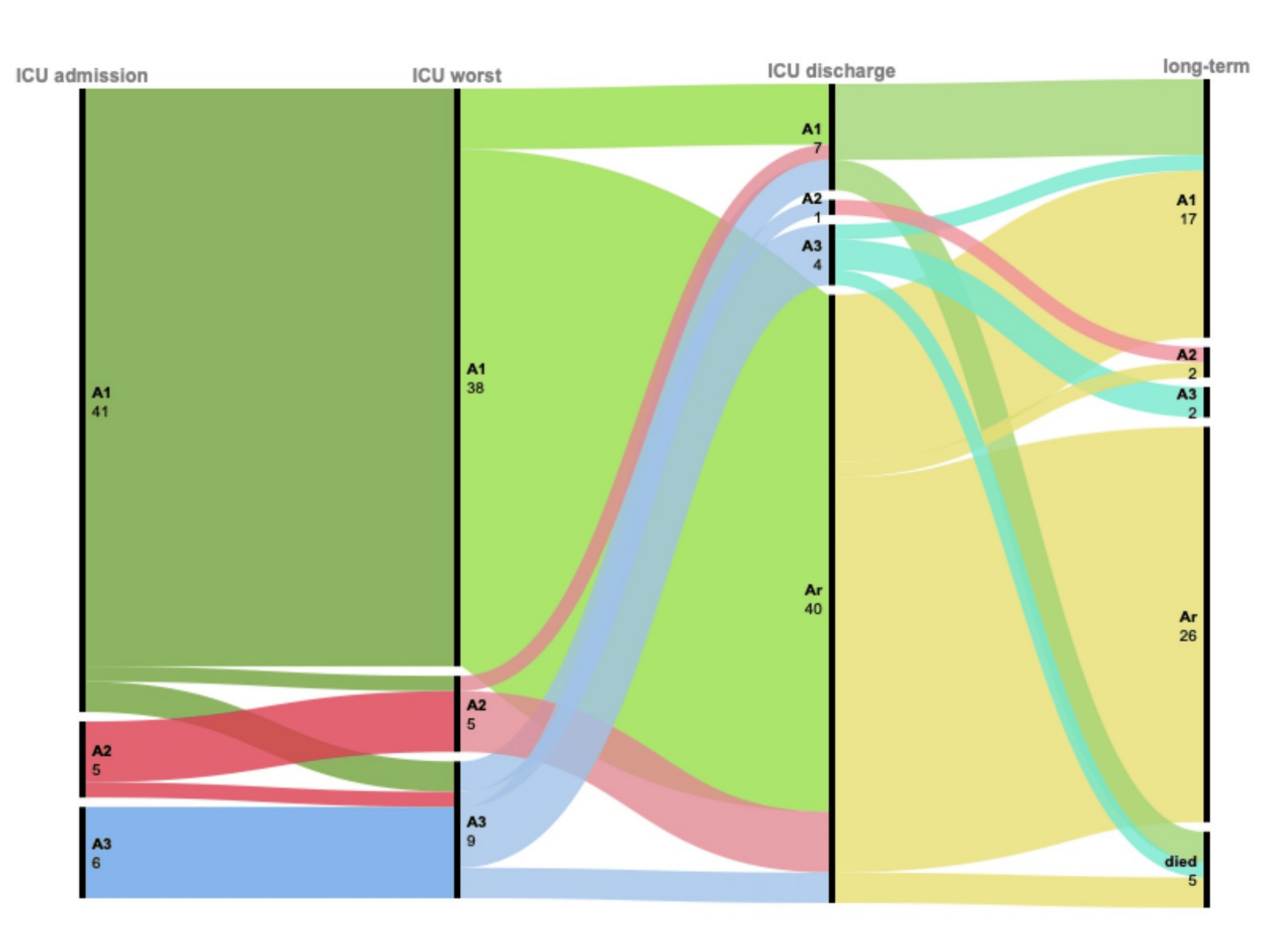
Alluvial diagram reporting the trajectory of AKI in early-AKI survivors Ar: AKI recovery; A1: AKI stage 1; A2: AKI stage 2; A3: AKI stage 3 Figure created by the authors with Microsoft Excel (Microsoft Corp., USA) and RAWGraphs (DensityDesign Lab, Studio Calibro, Studio InMagik)

The alluvial diagram (Figure 2) illustrates the trajectory of early-AKI survivors across four time points: ICU creatinine at admission, ICU worst creatinine, ICU creatinine at ICU discharge, and long-term creatinine value. Each column corresponds to a specific time point, and each colored flow shows how the patients move from one AKI stage to another over time. Most of the follow-up survivors start on AKI-1 (41 patients) stage. AKI severity tends to worsen during ICU stay from AKI-1 (A1) to AKI-2 (A2) and AKI-3(A3), and from A2 to A3. Thus, A3 becomes more prevalent in non-survivor patients, showing peak AKI severity. At ICU discharge, there is a general improvement in AKI status, and renal recovery (Ar) becomes the most dominant group (40 patients). Then, at long-term follow-up, a sustained recovery is observed in most cases, Ar remains high (26 patients), and the remaining patients subsequently evolved to AKI-1 during the follow-up period, the majority of whom had pre-existing chronic hypertension. Despite the limited number of patients with AKI-2 and AKI-3, partial renal recovery was observed at ICU discharge and persisted during the follow-up period. 

Patients’ outcomes

The ICU mortality was higher in early AKI than in no AKI (38% vs. 9%, p < 0.001). In early AKI, the ICU mortality increased with the KDIGO grade at admission, from 32% in AKI-1 to 44% in AKI-2 and 66% in AKI-3. In addition, mortality increased with the poor progression of AKI stage and use of RRT (up to 86% mortality in RRT-treated patients). Of the 52 early-AKI ICU survivors, two died during the hospital stay, and three died during the long-term follow-up period; therefore, 47 were alive 3.5 years after ICU discharge. 

**Figure 3:**
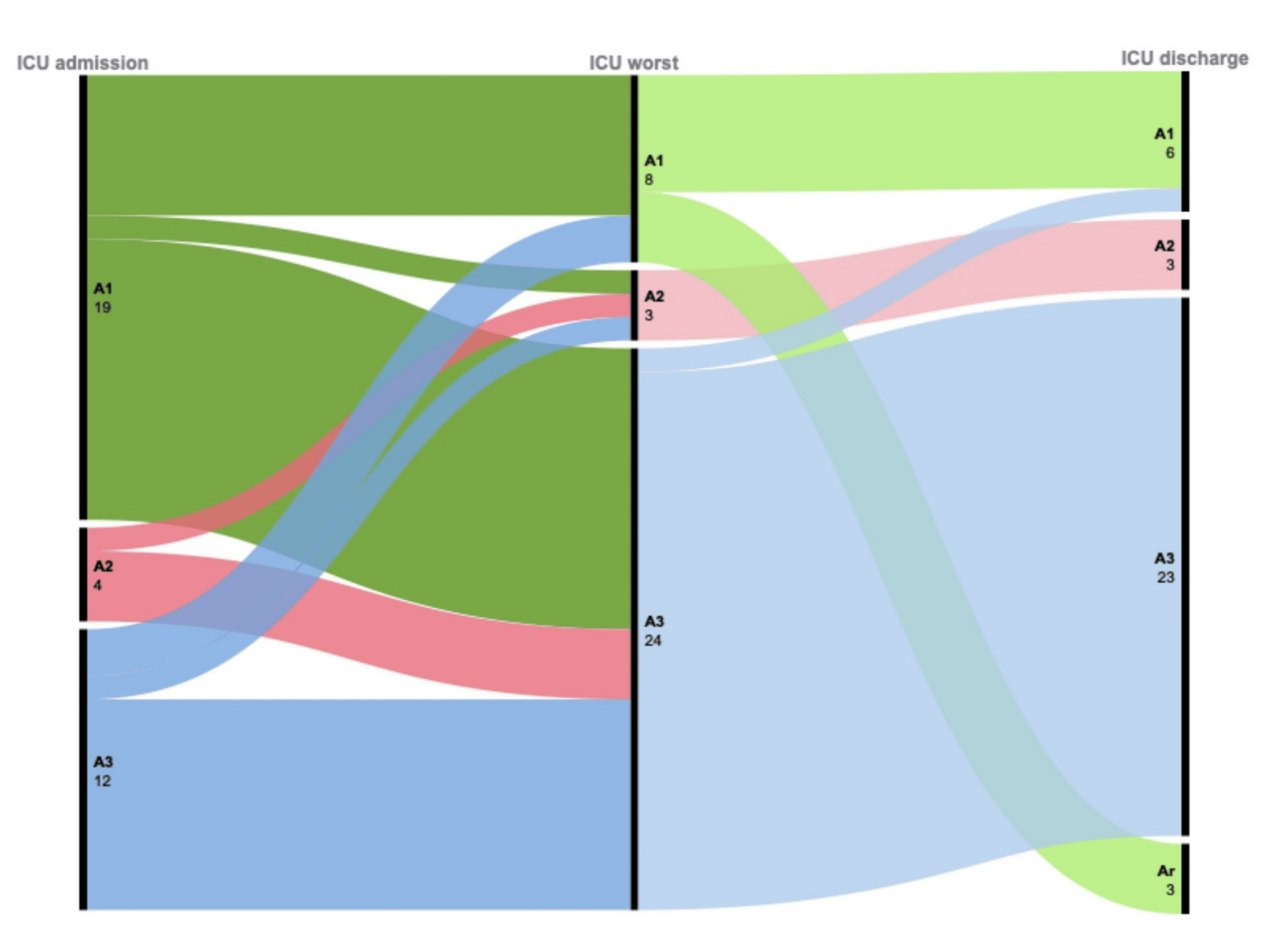
Alluvial diagram reporting trajectory in early AKI non-ICU survivors Ar: AKI recovery; A1: AKI stage 1; A2: AKI stage 2; A3: AKI stage 3 Figure created by the authors with Microsoft Excel (Microsoft Corp., USA) and RAWGraphs (DensityDesign Lab, Studio Calibro, Studio InMagik)

Figure 3 represents the trajectory followed by patients with early AKI during their ICU stay, up to the point of ICU death: Among the 60 AKI-1 patients, 19 died in the ICU, including 12 (63%) who progressed to AKI-3. In the nine AKI-2 patients, four patients died in the ICU following progression to AKI-3 in 75% (3/4), and among the AKI-3 patients, 12 died in the ICU (including nine (75%) who required RRT). 

**Figure 4:**
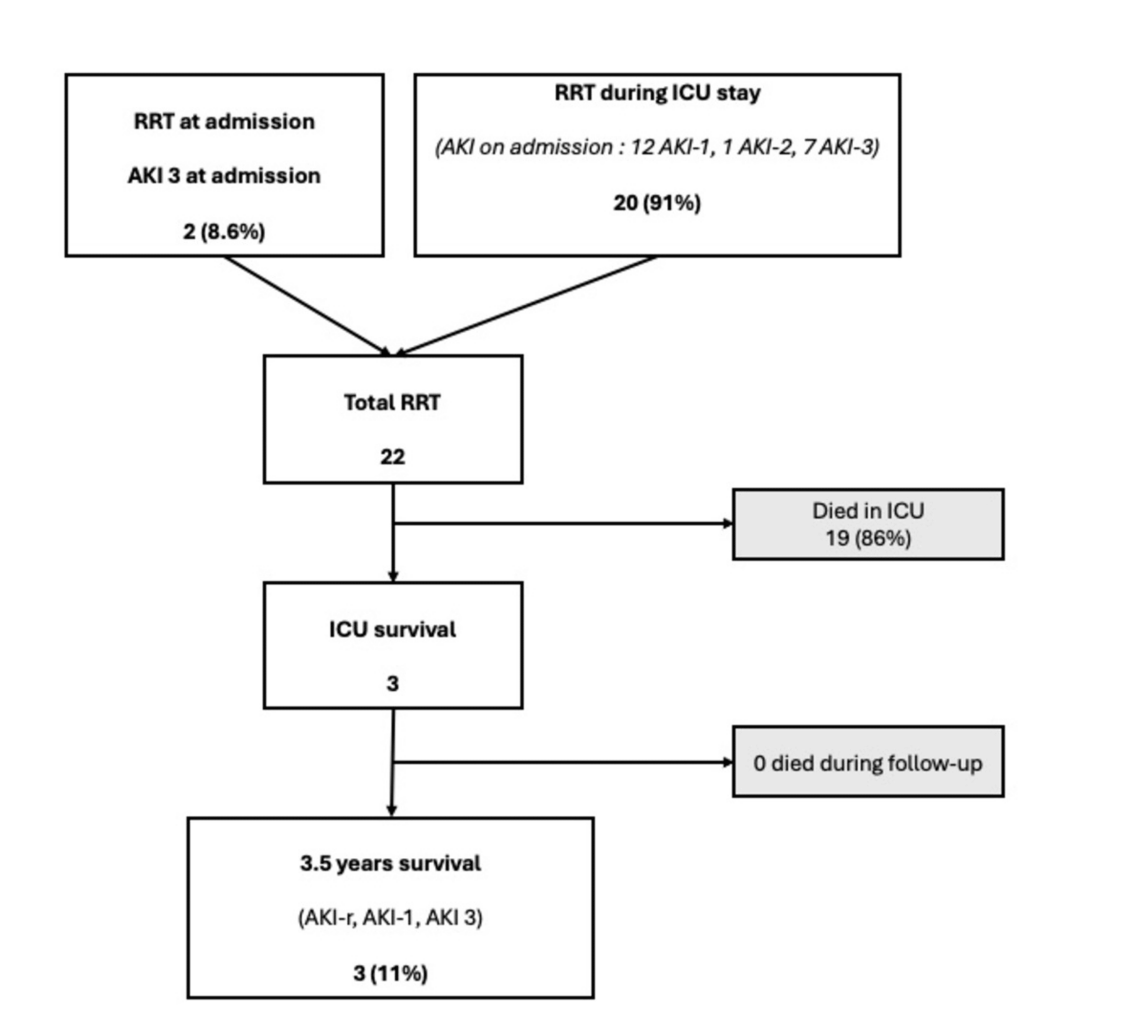
Long-term outcome (3.5 years) in patients receiving renal replacement therapy at any stage RRT: renal replacement therapy; AKI-1: AKI stage 1; AKI-2: AKI stage 2; AKI-3: AKI stage 3, AKI recovery Figure created by the authors with MS Word and PowerPoint (Microsoft Corp., USA)

Figure 4 represents the long-term outcomes for the 22 patients requiring RRT at any time during their stay: two (9%) patients received RRT at ICU admission, and 20 (91%) later during their ICU stay. Only three (11%) of these RRT patients survived and were discharged from the ICU. These three patients were alive at 3.5 years of follow-up and had sufficient recovery of renal function to discontinue dialysis; one patient admitted with AKI-A progressed to AKI-3, and from the two patients admitted with AKI-3, one of them completely recovered, and the other one evolved to AKI-1 during the follow-up period. 

## Discussion

The main findings of this study are that the long-term renal outcomes in early-AKI COVID patients admitted to the ICU were generally good, as most patients regained their renal function, except those who received RRT during their ICU stay. Mortality among early-AKI COVID patients was associated with RRT requirement at any time in the ICU, rather than the stage of AKI at admission. 

We observed that the incidence of AKI in COVID ICU patients was in line with published literature. In a meta-analysis [6], COVID-19 and AKI incidence ranged from 0.5% to 76.7%, and many factors may contribute to explaining this broad range of incidence, including different AKI definitions and the variable severity of patients hospitalized. For COVID-19 ARDS patients, the AKI incidence was reported at 44% at any time among COVID patients requiring both non-invasive and invasive mechanical ventilation [5]. In addition, Sitina et al. [11] observed that 6% of the patients without AKI on ICU admission developed AKI within the first 48 hours of ICU stay, and among the patient admitted with AKI, 30% experience deterioration of renal function during ICU stay and the change in creatinine within 48 hours of admission by more than 20% remained a significant predictor of 90 days ICU mortality. In a very large international database, McNicholas et al. [13] reported AKI within 48 hours of ICU admission in 21% of the ARDS COVID patients.

In our cohort examining ICU-COVID-19 patients of the first two waves, we observed an incidence rate of 36% of AKI on admission, with the majority (69%) experiencing the least severe form (AKI stage 1). Several factors likely contribute to AKI development at admission, including pre-admission poor oxygenation, dehydration due to digestive issues, fever, and decreased fluid consumption, along with the prior frequent use of nonsteroidal anti-inflammatory drugs. As commonly noted, patients exhibiting worsened renal function tended to have more comorbid conditions, such as metabolic syndrome (comprising diabetes, obesity, and hypertension), and were generally older, consistent with findings in other studies [5-7].

The progression of these patients appeared dichotomous. A favorable outcome was observed in AKI-1 stage patients, with complete recovery of renal function in 57% (34/60 patients) at ICU discharge; however, during the follow-up period, renal function deteriorated in nine patients, most of whom had pre-existing hypertension. Conversely, early-AKI-3 patients, who showed no renal function improvement, experienced a high ICU mortality rate of 66% (8/18 patients). Among those who required RRT, ICU mortality increased further to 78% (seven patients died from nine RRT).

Differences in outcomes are likely attributable to differences in severity at ICU admission, as most patients with unfavorable outcomes had severe organ dysfunction, ARDS, and hemodynamic instability, as measured by the SOFA score at admission. As a large epidemiological study [14] comparing AKI in COVID-19-related ARDS versus non-COVID-19-related ARDS, hemodynamic shock was found to be less frequent in the COVID-19-related ARDS group. Similar results were found in our cohort; the mean SOFA score remained relatively moderate, even in the presence of significant organ dysfunction such as AKI and respiratory failure. 

Our results help better characterize short- and long-term recovery from AKI. Most AKI-1 patients had good ICU outcomes and long-term recovery of renal function. It remains impossible to diagnose AKI stage 2 due to the small cohort size. More than half AKI-3 patients experienced deteriorating renal function and received RRT. We observed a significant relationship between the severity of ARDS (88% of AKI-3 on admission need mechanical ventilation and 33% need an ECMO during ICU stay) and the severity of AKI, due to higher intrathoracic pressure and higher inflammation. A secondary analysis of a multicenter observational study [15] found that the presence of AKI was associated with prolonged mechanical ventilation and increased mortality. Moreover, patients who developed early AKI have more severe ARDS compared to those without AKI. These findings are in line with our results, as we also observed that the worst trajectory of early AKI during ICU stay is an important predictor of mortality in COVID-19 patients. The mortality observed during the follow-up period was higher in the group evolving to AKI-3 (up to 77%) compared to patients evolving to AKI-1 (35%); this mortality was primarily at an early stage. 

The use of RRT in our cohort was 25%, similar to other studies [16,17]. The decision to initiate RRT follows established guideline criteria and avoids unnecessary early initiation [18,19]. In this specific group of early-AKI patients, RRT was most often started because of hypervolemia in cases of severe hypoxemia and no response to diuretic treatment. Among the RRT three-year survivors, renal recovery was sufficient to allow cessation of RRT, but many failed to fully recover, except one of them. Few studies have reported long-term outcomes in ICU COVID-19 patients presenting with early AKI, and despite the limited number of surviving patients in our study, we can observe a good recovery. Schmidt et al. [7] observed kidney outcomes following a 90-day follow-up in cases of mild to moderate COVID-19, noting no ongoing or progressive kidney sequelae. In a separate study with a two-year follow-up, Aklilu et al. [15] examined a cohort of 987 non-ICU COVID-AKI patients, reporting a faster deterioration of kidney function post-hospitalisation, which gradually diminished over two years compared to influenza-associated AKI and AKI from other illnesses. Notably, these studies encompassed patients who developed AKI at any stage, rather than focusing solely on AKI at admission. Gupta et al. [17] found a mortality rate of 63% during a short follow-up period of 60 days after ICU discharge, and in the survivors, 34% remained RRT dependent.

Our results are aligned with those of Seong Geung et al. [20], who found a similar trajectory for early AKI patients despite a brief follow-up period (30 days), suggesting that the onset and progression of AKI increase both mortality and length of ICU stay.

We reported the long-term consequences in patients who had recovered renal function at ICU discharge. While most of the patients maintained the renal function at the level observed at ICU/hospital discharge, a few patients improved their renal function, and 19% (9/47 long-term survivors) deteriorated their renal function during the follow-up period. These last patients were mostly patients with predisposing risk factors such as hypertension (5/9), suggesting that long-term monitoring of renal function should be proposed in this group of patients after hospital discharge. Indeed, the study by Harel et al. [21] reported that a post-AKI nephrologist follow-up was associated with better long-term renal outcomes and reduced mortality. Unfortunately, this was not always performed in these patients due to the ongoing pandemic that limited outpatient clinic activities.

We also acknowledge several limitations. First, our study is a single-center study, which may limit external validity. However, this favored the completeness of data collection. Second, the small sample size, especially for certain relevant subgroups, reduces the statistical power. The retrospective design precludes establishing causality between RRT and outcomes. Third, identifying therapeutic options (steroids, anti-IL-6, etc.) that might have improved long-term outcomes was not analyzed in our study concentrated on the initial COVID waves. Fourthly, the reliance on plasma creatinine values rather than clearance measurements may have led to slight overestimations of renal recovery; also, in the same way, the back calculation baseline SCr could be a potential source of bias in AKI classification. Fifth, because of the incomplete biological characterization of AKI events at admission (proteinuria, baseline renal function, and urine sediment analysis), the finding may not generalize to later COVID waves due to evolving variants, vaccination impacts, and the use of steroids. Sixth, we performed a three-and-a-half-year follow-up only of the patients who presented with early AKI, and sometimes used telephone interviews as an alternative means of data collection, which represents a potential source of information bias. We do not have long-term data on the no-AKI COVID patients. 

## Conclusions

Early AKI is common among ICU patients hospitalized for COVID-19 acute hypoxic respiratory failure and is linked to longer ICU LOS. Mortality in this patient is more correlated with the requirement for RRT during the ICU stay than with the AKI stage at ICU admission. Identifying early risk factors may help stratify patients and guide follow-up after hospitalisation.
